# Corpus Colossal: A Bibliometric Analysis of Neuroscience Abstracts and Impact Factor

**DOI:** 10.3389/fnint.2019.00018

**Published:** 2019-07-03

**Authors:** William M. Kenkel

**Affiliations:** Neuroscience Institute, Georgia State University, Atlanta, GA, United States

**Keywords:** neuroscience, brain, bibliometric, meta-research, information science

## Abstract

A field's priorities are reflected by the contents of its high-impact journals. Researchers in turn may choose to pursue research objectives based on what is believed to be most highly valued by their peers. However, these assessments of the field's priorities are often subjective, owing to a lack of formal quantification of high-impact journals' contents. By compiling a corpus of abstracts from within the field neuroscience, I was able to analyze which terms had differential frequencies between 13 high-impact and 14 medium-impact journals. Approximately 50,000 neuroscience abstracts were analyzed over the years 2014-2018. Several broad trends emerged from the analysis of which terms were biased toward high-impact journals. Generally speaking, high-impact journals tended to feature: genetic studies, use of the latest and most sophisticated methods, examinations of the orbitofrontal cortex or amygdala, and/or use of human or non-mammalian subjects. Medium-impact journals tended to feature motor or cardiovascular studies, use of older methods, examinations of caudal brain regions, and/or rats as subjects. This approach also allowed for the comparison of high-impact bias among: brain regions, methods, neurotransmitters, study species, and broad themes within neuroscience. A systematic approach to the contents of high-impact journals offers the field an objective view of itself.

## Introduction

A journal's impact factor is determined by the number of citations received relative to the number of articles published. The prospect of publication in high impact journals is rewarding to neuroscientists, and leads to activation of the nucleus accumbens in an impact factor dependent manner (Paulus et al., 2015). This is likely because within the culture of academic research, the contents of high-impact journals are taken as a proxy for the interests and priorities of the field. Researchers use their sense of the field's priorities to dictate their own research decisions, as well as in evaluating others' work. These impressions are shaped by experience, training, and social conditioning, often without much systematic analysis.

Previous efforts have identified the 100 most-cited papers in neuroscience (Yeung et al., 2017a) or identified factors associated with high impact authorship, such as age (Sugimoto et al., 2016) or gender (West et al., 2013; van den Besselaar and Sandström, 2016; Shen et al., 2018). Among high-impact neuroscience and multidisciplinary journals, women authors are persistently underrepresented (Shen et al., 2018). Scholars from younger generations receive less recognition despite publishing in better journals (Sugimoto et al., 2016). Across a researcher's career, the number of citations peak in the early years then continually decline, while the impact factor of journals they publish in remains fairly stable (Sugimoto et al., 2016). Publication in high-impact journals is aided by the inclusion of senior co-authors who have published in those same journals previously, an effect which is referred to as a “chaperone effect” and is especially prominent in biomedical fields such as neuroscience (Sekara et al., 2018). These patterns are important to understand because there is also a “Matthew effect” acting on research careers (Merton, 1968; Sugimoto et al., 2016; Bol et al., 2018), such that early advantages and success accumulate and increase the likelihood of access to further recognition, citation and funding success. Thus, publishing in a high-impact journal early in one's career is likely to improve career trajectory in ways that produce large benefits later on.

The most complete analysis of neuroscientific publication trends analyzed work from 2006-2015 (Yeung et al., 2017b). This work, by Yeung et al., analyzed the patterns in citations among individual articles and observed a shift of focus from general brain imaging terms to cellular, molecular and genetic terms over the study period. The purpose of the present work was to take a bird's-eye view of recent neuroscience publications and determine patterns in the terms that are differentially used in high- vs. medium-impact journals.

## Methods

Abstracts were gathered from 13 high-impact and 14 medium-impact journals, creating two corpora spanning January 2014 to December 2018. Journals were selected according to the following criteria: (1) each journal must have a broad scope within the field of neuroscience, that is, they could not be limited to a single sub-discipline, method, or species; and (2) journals must follow conventional practices for abstracts and issue composition. Thus, in addition to many journals being excluded for being too narrowly focused, three high-impact journals in particular were left out: *Brain and Behavioral Sciences* was excluded for being too theoretical and dissimilar to other journals' content; *Trends in Neuroscience* was excluded for having too brief abstracts; and *Progress in Brain Research* was excluded for having non-conventional issue composition. For three high-impact journals with especially broad, transdisciplinary scopes (*Science, Nature*, and *Nature Communications*) a further criterion was applied such that each abstract was scanned for occurrences of a neuroscience-related keyword and only included if at least one such keyword was present. The list of high- and medium-impact journals is shown in Table 1; the list of keywords indicated neuroscience relevance within the three broad scope high-impact journals is shown in Table 2. Truly low-impact journals were avoided as being overly niche. For medium-impact journals, impact factor ranged from 1.26 to 5.97, with a median of 2.97. For high-impact journals, impact factor ranged from 10.85 to 41.58, with a median of 14.67. In a separate analysis, the analyses were run with the high-impact journals *Nature Reviews Neuroscience* and *Annual Review of Neuroscience* removed to gauge the effect of review-only journals.

**Table 1:** Journals included in the present analyses.

**High-impact journals:** *Acta Neuropathologica, Annual Review of Neuroscience, Biological Psychiatry, Brain, Cell, Molecular Psychiatry, Nature, Nature Communications, Nature Neuroscience, Nature Reviews Neuroscience, Neuron, Progress in Neurobiology*, and *Science*. **Medium-impact journals:** *BMC Neuroscience, Brain and Behavior, Brain Research, Brain Research Bulletin, Brain Structure and Function, European Journal of Neuroscience, Frontiers in Neuroscience, Journal of Neurophysiology, Journal of Neuroscience, NeuroReport, Neuroscience, Neuroscience Bulletin, Neuroscience Letters, Neuroscience Research*.

**Table 2:** Neuroscience-related keywords for screening science, nature, and nature communications articles.

“acetylcholine,” “AMPAr,” “amygdala,” “autism,” “axonal,” “axons,” “brain,” “brainstem,” “cerebellum,” “dendrite,” “dendrites,” “dopamine,” “endorphins,” “GABA,” “glutamate,” “grid fields,” “gyrus,” “hippocampus,” “hypothalamus,” “long-term potentiation,” “long-term depression,” “memory consolidation,” “myelin,” “myelination," “neural,” “neuron,” “neurons,” “neuroscience,” “neurotransmitter,” “NMDAr,” “oxytocin,” “place fields,” “progesterone,” “serotonin,” “sulcus,” “synapse,” “synaptic,” “thalamus,” and “vasopressin.”

Abstracts were harvested from PubMed using a combination of the *PubMedWordCloud*, and RISmed packages for R, along with direct scraping of ncbi.nlm.nih.gov/pubmed. Citations of each journal article was collected using the rcrossref package for R. All text was then converted to lower case, but for the sake of clarity will be shown in its most-common form in the following text and legends. After removing numbers, punctuation, and commonly used English stop words (e.g., “a,” “is,” “the”), each corpus was cleared of generic research terms (e.g., “effect,” “group,” “increased”). A further set of terms was then removed for being either spurious or accidents of publication (e.g., “copyright,” “Ireland,” “university”). Various typographical errors were addressed on an as-needed basis, which consisted chiefly of incorrectly conjoined words (e.g., “patientderived”), reconciling certain plurals (e.g., combining “tumor” and “tumors”), or resolving discrepant spellings between American and British English.

Each corpus was then analyzed using the *tidytext* and *tm* (text mining) packages for R. The R scripts used for analysis and raw data are attached as Supplementary Files. Incidences of each unique term were calculated and then compared between high- and medium-impact journals, controlling for each corpus' overall word count. Three primary measures were collected from each term in the resulting corpus: (1) the log odds ratio, defined as the log of the ratio of the proportion of instances of a given term (adjusted for the differing sizes of the high- and medium-impact journal contents); (2) the impact rate, defined as the sum of relevant impact factors for each instance of a given term divided by the total number of instances of the term; and (3) the rate of citations garnered, defined as the total number of citations for each article to use a given term divided by the total number of instances of the term. Thus, a higher log odds ratio indicates a given term to be favored among the group of high-impact journal abstracts listed in Table 1; a higher impact rate indicates a given term was favored by higher impact journals generally; and a higher citation rate indicates a given term was featured in articles that garnered more citations. In order to be included in the final analyses, a term had to be used more than 10 times per year.

Secondary analyses were carried out comparing (1) study organisms, (2) brain regions, (3) neurotransmitters, (4) methodological approaches, and (5) broad themes within neuroscience, comparing in each case the log odds ratio of occurrence in medium- vs. high-impact journals. Each broad theme from within the larger field of neuroscience was assigned several keywords intended to be specific to that particular theme, as shown in Table 3. Each methodological approach was first evaluated for the specific term that was most frequently used (e.g., “optogenetics” rather than “optogenetically”). The various conjugations of these methodological terms did not meaningfully differ in terms of odds ratio of occurrence.

**Table 3:** Categories and their associated terms.

***Alzheimers:** alzheimers, amyloid, APOE, dementia, plaque, tau **Cellular:** cellular, extracellular, intracellular, nuclear, trafficking, translocation **Comparative:** comparative, evolutionary, phylogenetic, species **Depression:** anxiety, depression, depressive, MDD **Drug abuse:** abuse, addiction, drug, drugs, reward **Epigenetics:** CPG, epigenetic, histone, methylation **Learning and Memory:** conditioned, consolidation, extinction, habituation, learning, memory, retrieval, retention **Molecular:** kinase, pathway, phosphorylation, subunit **Microbiome:** gut, microbiota **Neurogenesis:** fate, neurogenesis, progenitor, progenitors, stem **Neuroimmunology:** cytokine, cytokines, inflammation, interleukin, microglia, pro-inflammatory, neuro-inflammation **Plasticity:** LTP, plasticity, potentiation, voltage **Social:** empathy, maternal, social **Stress:** adrenal, corticosteroid, corticosterone, cortisol, HPA, stress **Synaptic:** axonal, dendritic, post-synaptic, pre-synaptic, receptor, synaptic*

## Results

**Figure 1:**
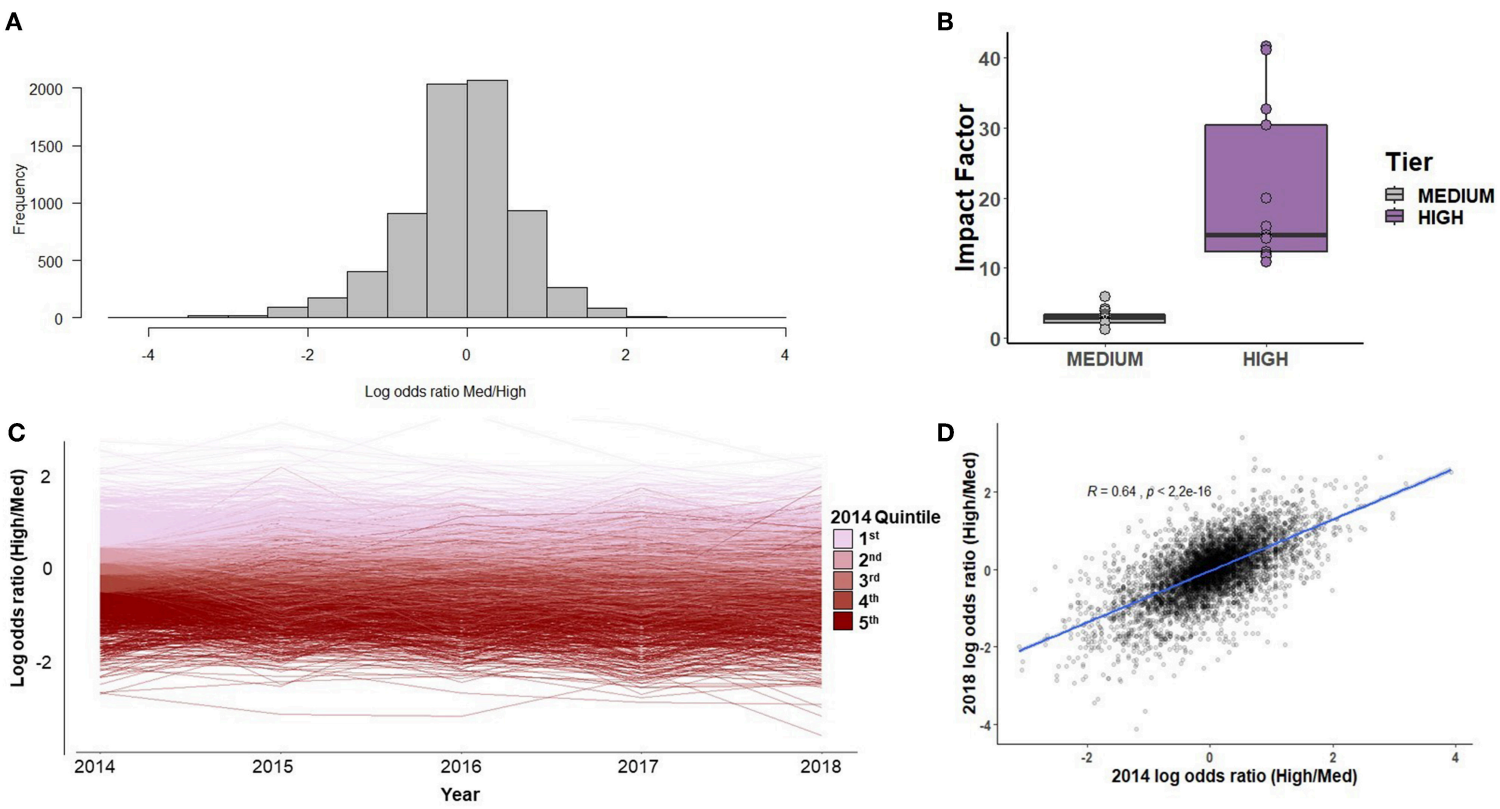
**(A)** The histogram of terms' log odds ratios. A negative log odds ratio indicates that a term was used more frequently by medium-impact journals. **(B)** The distribution of impact factors among the 13 high- and 14 medium-impact journals selected for comparison. The two outlying high points among the high-impact journals were Nature and Science. **(C)** The consistency of log odds ratios over time, color-coded by log odds ratio quintile as of 2014. **(D)** The correlation of log odds ratios between 2014 and 2018.

A total of 15,461 abstracts were gathered from high-impact journals and 34,526 abstracts from medium-impact journals. The distribution of impact factor scores among the two categories of journals is shown in Figure 1A. The distribution of terms' log odds ratios was roughly symmetrical, with slightly more terms differentially preferred by medium-impact journals (Figure 1B). There was a moderate degree of consistency in terms of log odds ratio across the study period, such that terms in the highest quintile in 2014 tended to remain in that slightly less than half (43.5%) of the top quintile terms in 2014 remained in the top quintile in 2018 (Figure 1C). The overall correlation of a term's log odds ratio in 2014 to that of 2018 was R^2^ = 0.41 (Figure 1D). For the sake of comparison, a separate analysis was conducted comparing 2018 results to those of 2010 (in which case the journals NeuroReport and Brain and Behavior were excluded, as they had not yet been founded). In this analysis, a given term's score in 2018 was less well predicted by its 2010 score (R^2^ = 0.28).

**Figure 2:**
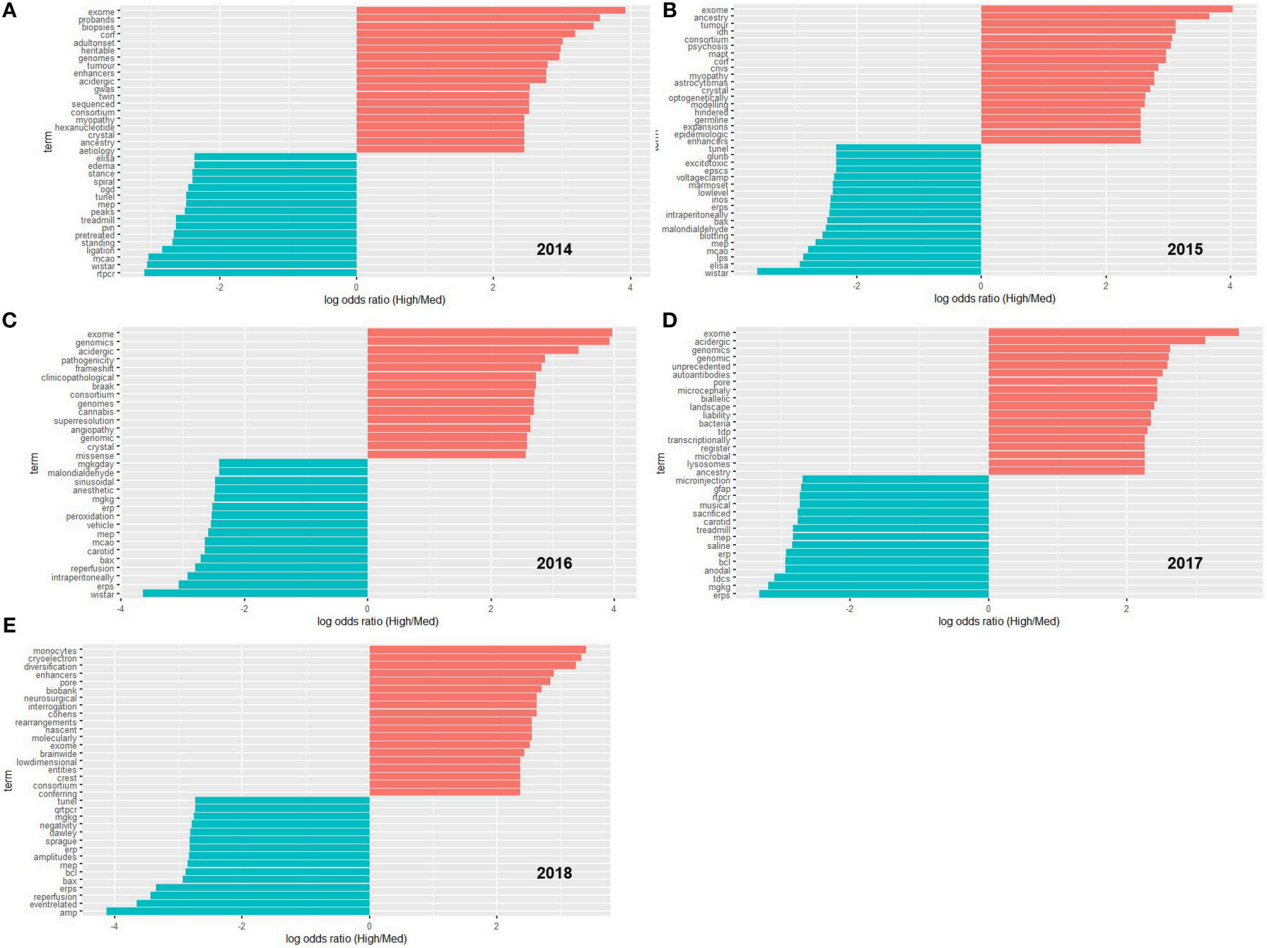
The 15-most biased terms for high- and medium-impact journals on a yearly basis, from 2014 **(A)** to 2018 **(E)**. Common English stop words and common terms of research/publication were removed. In order to be included, a term had to be used at least 10 times.

**Figure 3:**
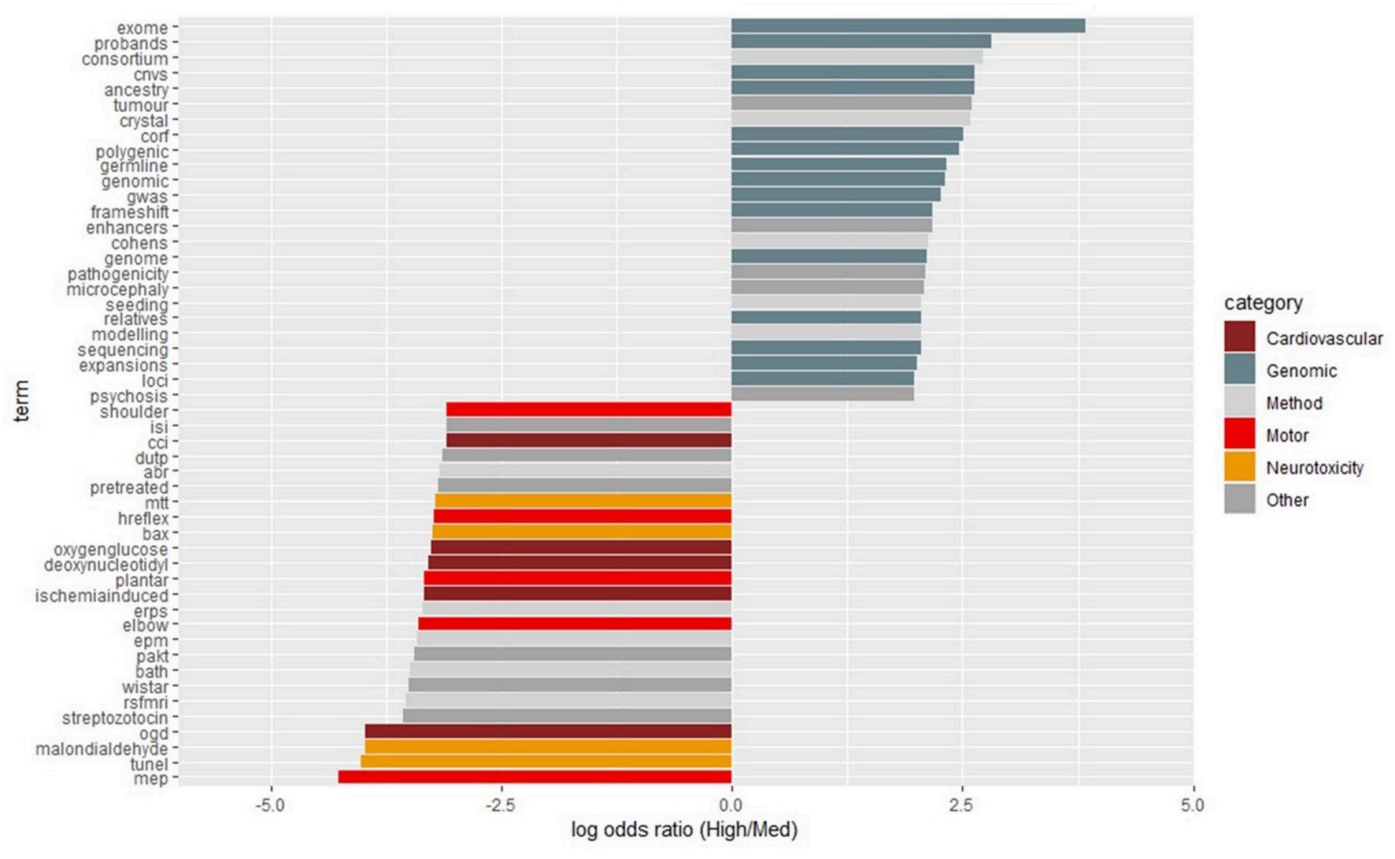
The 25-most biased terms for high- and medium-impact journals across the entire study period from 2014-2018. Terms were then categorized and color-coded as shown in the legends to the right. Common English stop words and common terms of research/publication were removed. In order to be included, a term had to be used at least 50 times.

On a year-by-year basis, terms scoring in the top 15 most differentiated (biased either toward high- or medium-impact journals) are shown in Figure 2. Over the entirety of the 5-year period, the terms scoring in the top 25 most differentiated were categorized into themes as shown in Figure 3.

Secondary analyses were conducted using the full complement of journals across the 5-year study period, comparing study organisms (Figure 5), brain regions (Figure 6), neurotransmitters (Figure 7), methodological approaches (Figure 8), and broad themes within neuroscience (Figure 9). In the cases of study organism, neurotransmitter and approach, the size of each dot represents the total number of instances each term occurred. For instance, rats were found to be commonly used and associated with medium-impact journals, while *C. elegans* was found to be uncommonly used and associated with high-impact journals.

## Discussion

Several broad themes emerged from the differential use of terms between medium- and high-impact journals. Throughout the study period of 2014 to 2018, there was a clear premium placed on genetic studies, as indicated by the high log odds ratios for such terms as shown in Figure 3. Indeed, the majority of high-impact biased terms were related to genetic/genomic analyses. On the other hand, terms related to cardiovascular function, motor function, and neurotoxicity tended to be biased toward medium-impact.

The abstracts of medium-impact journals tended to be more specific in terms of their methodology. The majority of specific neurotransmitters had negative odds ratios, as did many methodological approaches. Terms such as “*EPM*” (elevated plus maze), “*MEP*” (motor-evoked potential), and “*tunel*”—a marker of apoptosis, all spoke to a more methodologically detailed approach to abstract construction in medium-impact journals. Similarly, details of dosage, such as “*intraperitoneally*,” “*mg/kg*,” “*pre-treated*,” and “*vehicle*” were all skewed toward medium-impact journals. It is worth pointing out that many of the approaches favored by medium-impact journals also share the qualities of being widely accessible, long-established, and having low barriers to entry. For instance, the relatively ubiquitous event-related potential (“*event-related*,” “*ERP*,” and “*ERPs*”) was consistently among the most medium-impact based. Another trend among the medium-impact journal abstracts was the high frequency of rat strain names. “*Wistar*” and “*Sprague Dawley*” consistently featured in the top 15 most differentiated terms in favor of medium-impact journals. Among study organisms, the most frequent terms were rats and mice, although the phrasing of abstracts with regards to human subjects can be quite broad, and thus we are left without a direct comparison of the percentage of usage of each organism. The most frequent methodology term, “*behavior*” was equally used by both medium- and high-impact journals.

The analysis of research methodologies reinforced the pattern of genetic pre-eminence, with “*GWAS”* being the most high-impact biased term. Beyond that was a trio of terms relating to new, challenging and expensive approaches: “*transcriptome,”* “*RNA-seq*,” and “*chemogenetic*.” Without the inclusion of Journal of Neuroscience in the medium-impact group, “*optogenetics*” and “*chemogenetics*” were both among the most high-impact biased terms.

The overall pattern that emerges is that of a *U*-shaped curve if impact were the y-axis and a continuum of translatability were the x-axis. High-impact journals tended to feature more humans, as well as zebrafish, *C. elegans* and drosophila. Mammalian models, particularly rats, are left in the trough of this curve. To wit, within the “stress” theme, “*cortisol*” (the human glucocorticoid) had a higher log odds ratio than “*corticosterone*” (the rodent analog), 0.52 vs. −0.89, respectively. The issue of translatability is difficult to clearly define as an axis, and future work would benefit from a system that could distinguish work done in clinical patients, work done in healthy humans, and work done in the various animal models of clinical conditions.

DTI, MEG, and EEG were all toward the low end of the distribution of log odds ratios compared to other methodologies (Figure 8). This pattern is somewhat in contrast with Yeung et al.'s findings covering 2006-2015 (Yeung et al., 2017b), which identified “DTI” and “fractional anisotropy' as high-impact terms (−1.89 and −0.27 log odds ratio in the present work, respectively). This is likely due to neuroimaging having its own relatively high-impact journals that cater to it as a sub-field, such as: Human Brain Mapping, Neuroimage, and Biological Psychiatry: Cognitive Neuroscience and Neuroimaging. Thus, neuroscience may already be too large to consider as a unified field any longer. Sub-fields of neuroscience would surely benefit from analyses specifically tailored to their shifting research interests and methods.

“*CCI*” (chronic constrictive injury), “*edema*,” “*MCAO*” (middle cerebral artery occlusion), “*oxygen-glucose*,” “*OGD*” (oxygen-glucose-deprivation), “*reperfusion*,” and “*stenosis*” were terms biased toward medium-impact journals that all pertain to the cardiovascular pathology of stroke. These terms were spread across several medium-impact journals, which suggests a broad pattern. Indeed, previous analysis of neuroscience articles from 2006-2015 also identified ischemic stroke as having consistently low citation impacts, along with multiple sclerosis and intracerebral hemorrhage (Yeung et al., 2017b).

Among neurotransmitters, most terms were biased toward medium-impact journals, with only “*oxytocin*” and the “*AMPA*” glutamate receptor showing a substantially positive log odds ratio. On the other hand, themes were mostly biased toward high-impact journals. The pattern among themes remained similar when the threshold for term inclusion was dropped from 10 instances/year to 10 instances across the entire 5-year span, with the notable exception of the Microbiome theme, which increased. Within Microbiome, the lowered threshold led to the inclusion of “microbiome,” “germ-free,” and “microbial,” which each had log odds ratios >1.46. The microbiome theme did especially well when considered by citation rate, as it garnered substantially more citations than other themes. Among brain regions, there was a generally caudal-rostral progression in terms of log odds ratio, though with several exceptions. As expected, “cortex” was widely used, “*hippocampus*” was the most commonly used specific region, and “*orbitofrontal*” (cortex), a region almost exclusively studied in humans, the most high-impact biased.

**Figure 4:**
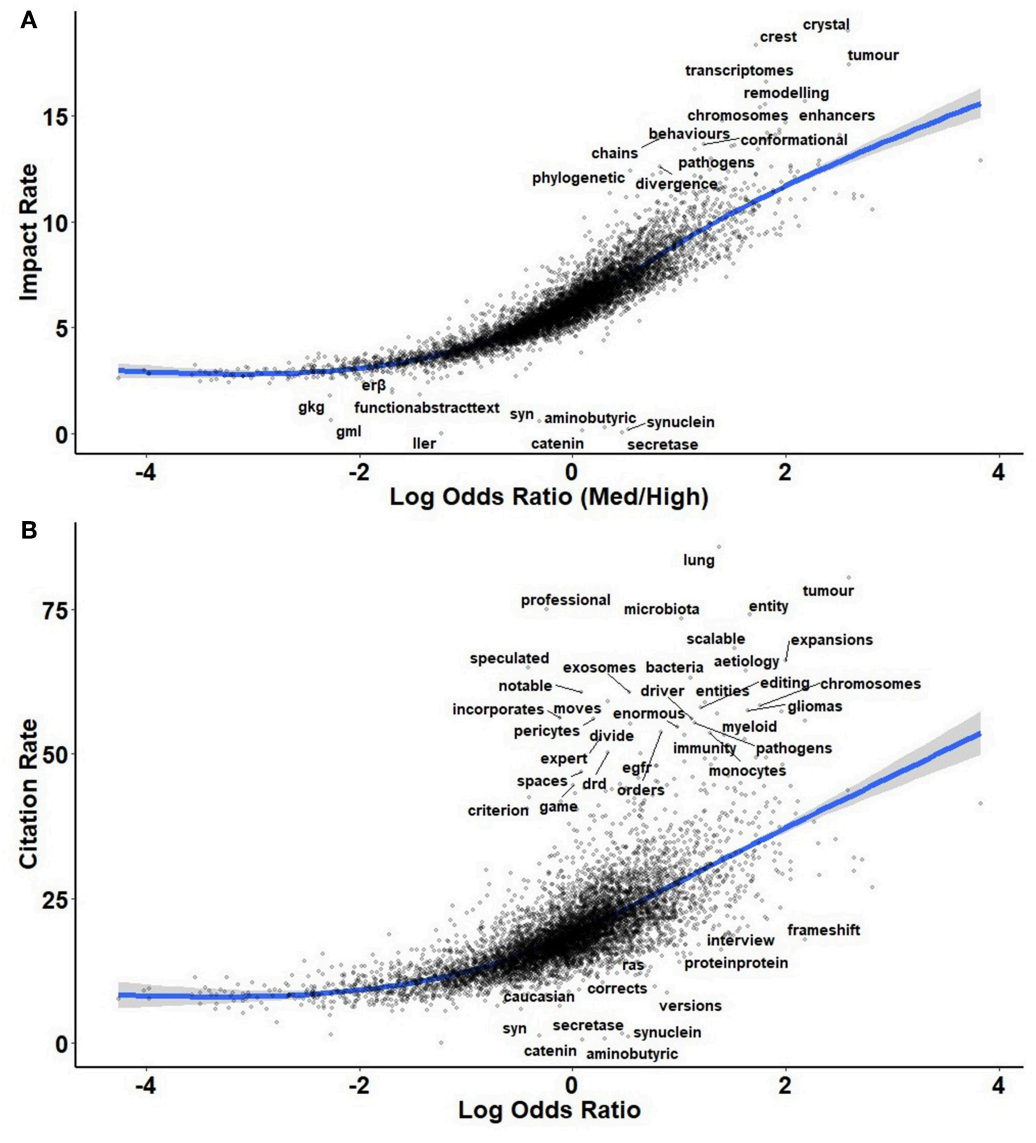
Plots of log odds ratio compared to the cumulative impact factor across each instance of a term **(A)** and to the number of citations per instance used **(B)** throughout the study period, 2014-2018. Terms with large residuals from the trendline are noted.

**Figure 5:**
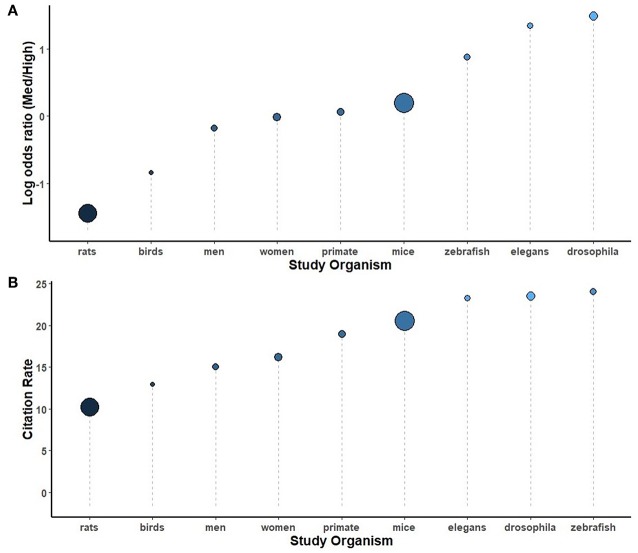
A comparison of study organisms sorted by log odds ratio **(A)** and by the number of citations per instance used **(B)** throughout the study period, 2014-2018. The size of each circle is proportional to the number of instances each term was used, from birds (145) to mice (6794).

**Figure 6:**
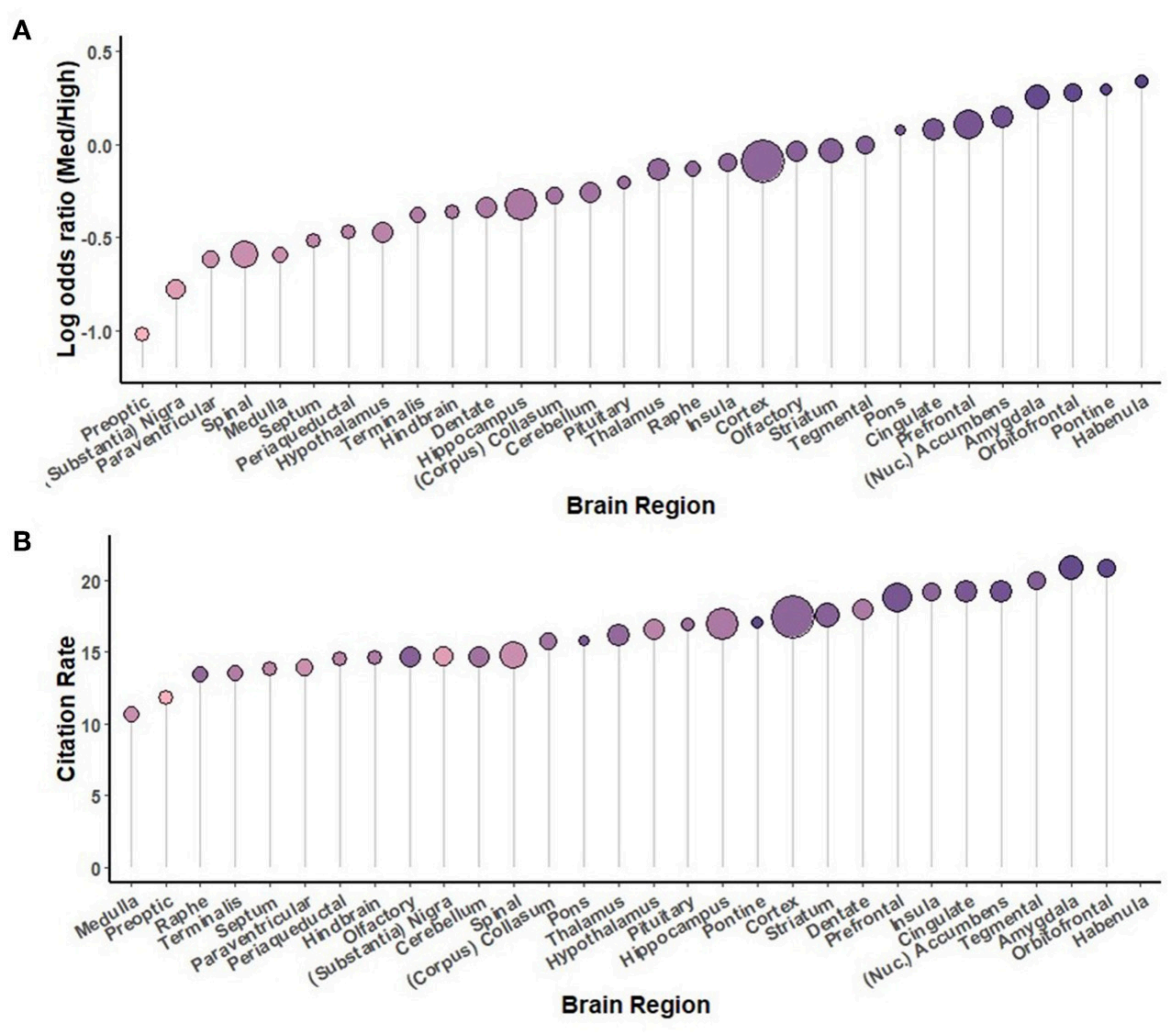
A comparison of brain regions sorted by log odds ratio **(A)** and by the number of citations per instance used **(B)** throughout the study period, 2014-2018. The size of each circle is proportional to the number of instances each term was used, from pons (76) to cortex (8291).

**Figure 7:**
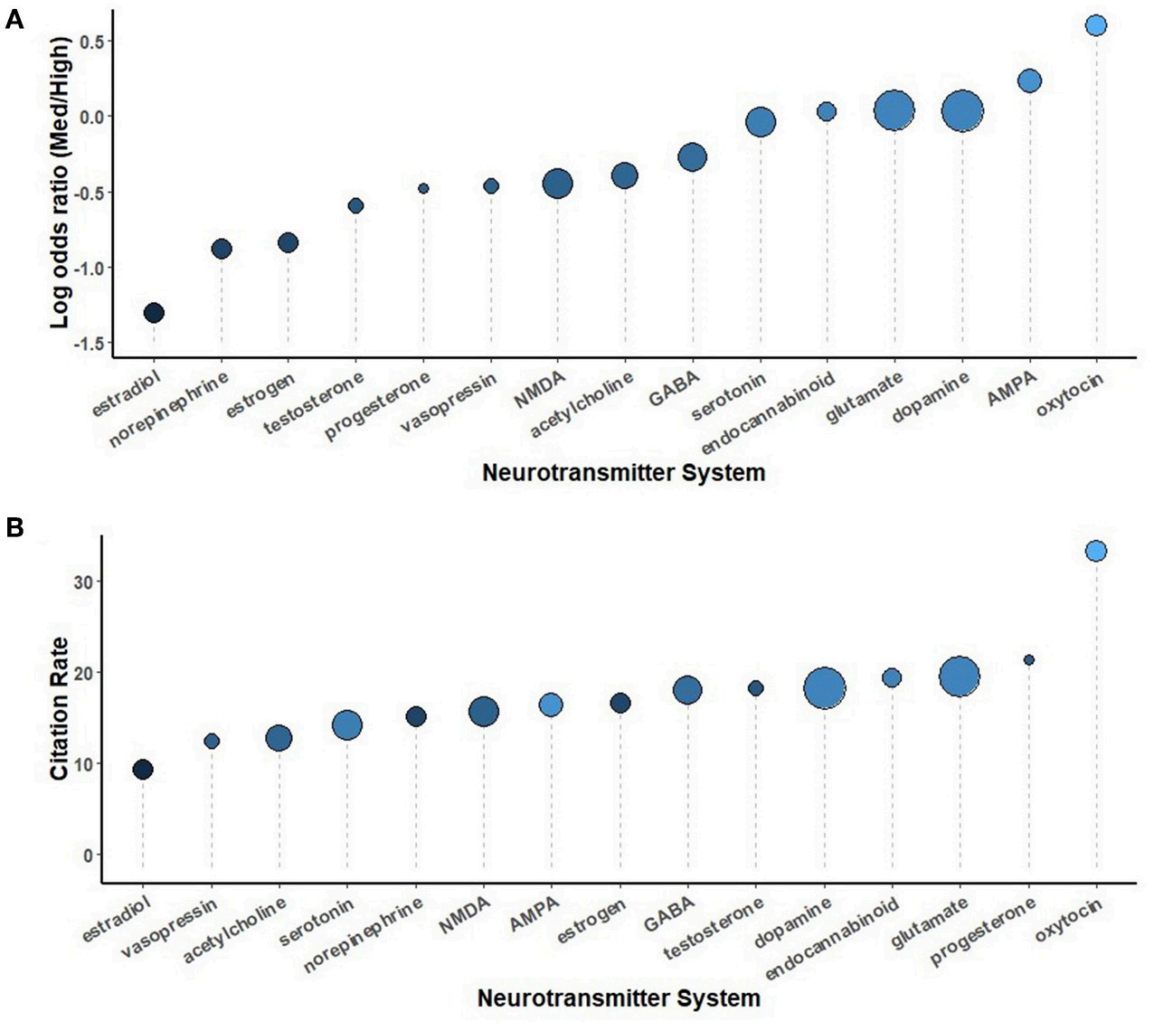
A comparison of neurotransmitters sorted by log odds ratio **(A)** and by the number of citations per instance used **(B)** throughout the study period, 2014-2018. The size of each circle is proportional to the number of instances each term was used, from progesterone (81) to dopamine (1843).

**Figure 8:**
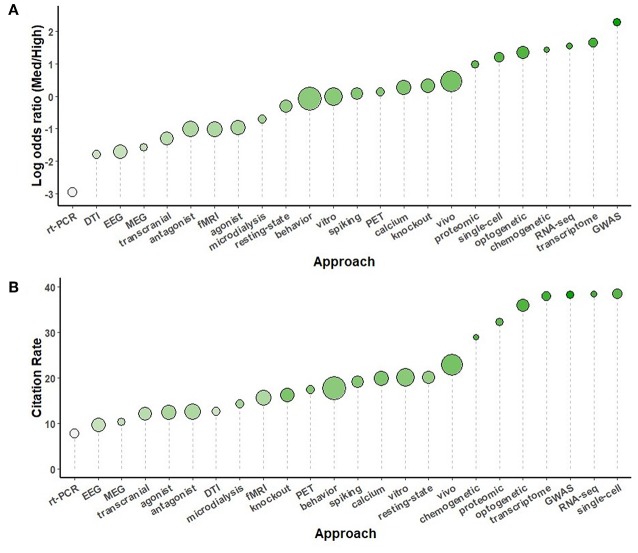
A comparison of methodological approaches sorted by log odds ratio **(A)** and by the number of citations per instance used **(B)** throughout the study period, 2014-2018. The size of each circle is proportional to the number of instances each term was used, from chemogenetic (93) to behavior (4369).

**Figure 9:**
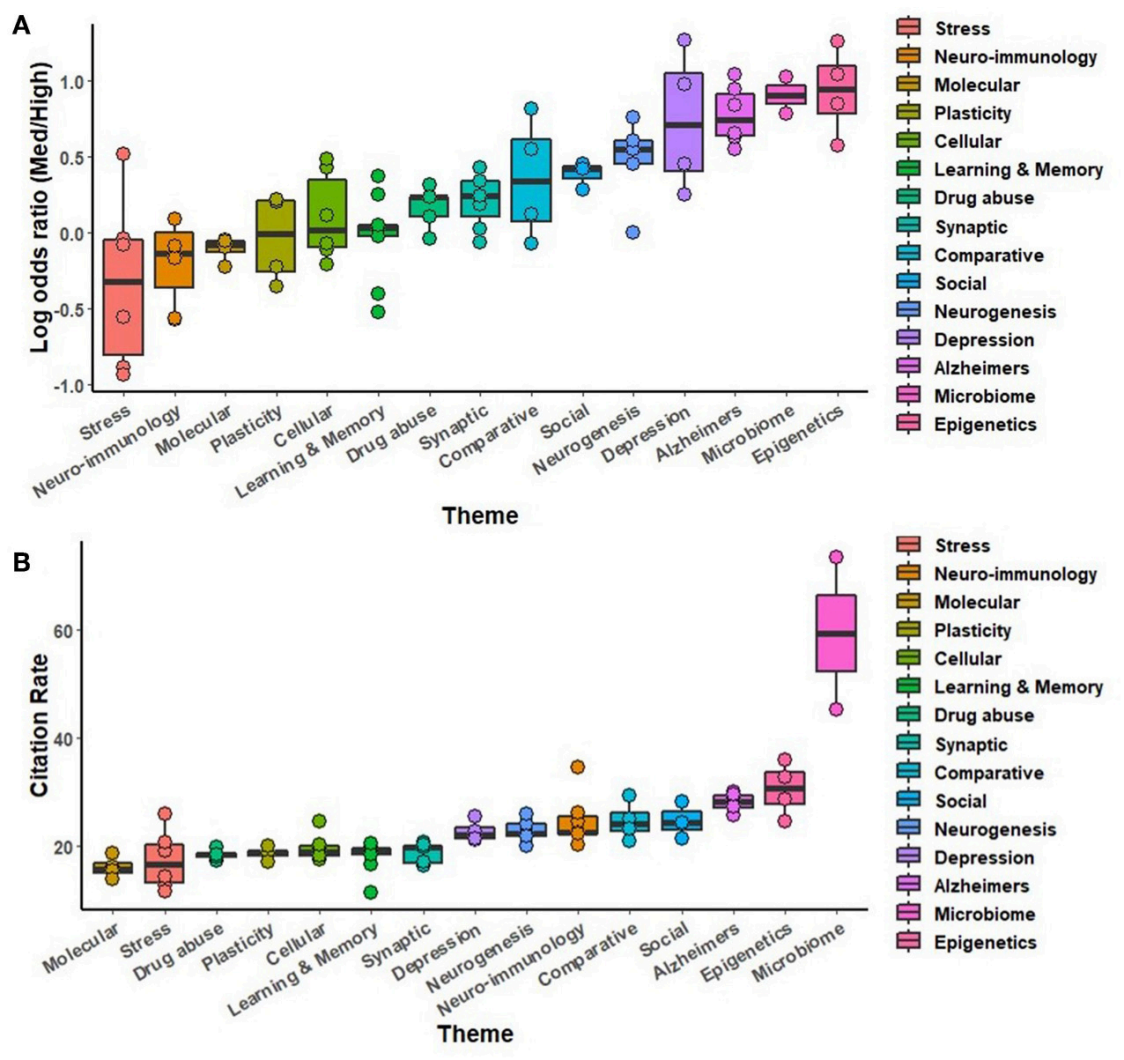
A comparison of broad thematic collections sorted by log odds ratio **(A)** and by the number of citations per instance used **(B)** throughout the study period, 2014-2018. Each theme consists of several related terms, as specified in Table 3.

By and large, the pattern of log odds ratios remained the same when impact was considered continuously (Figure 4A) and resembled the pattern of citations (Figure 4B). Were there terms infrequently used by high-impact journals yet still receiving of high rates of citations, such topics would be interesting as potentially over-looked by mainstream neuroscience. Unfortunately, few such candidates emerged, among them: “*tumor*,” “*lung*,” and “*microbiota*.” The microbiome theme also garnered substantially more citations than other themes. When carrying out curated comparisons (Figures 5–9), the patterns of terms' rate of citations collected tended to resemble those of the log odds ratios. To a certain extent, this correlation between citations and high-impact journal contents is tautological, though what is unknown is how much each factor defines the other. Are high-impact journals successful because they carry the most interesting research, or are research interests shaped by the contents of high-impact journals via authority bias?

Some of neuroscience's highest-impact journals consist entirely of review articles. Interestingly, even with these reviewonly journals removed, methodological details (e.g., dosage, administration route, control conditions) were still less likely to be found in high-impact journals. Indeed, the list of most differentiated terms was essentially identical between the original and review-removed datasets (data not shown). This suggests that the reviews introduced little in the way of rare or unique terms, which should perhaps be expected from reviews.

Previous work by Yeung et al. identified “autism,” “metaanalysis,” “functional connectivity,” “default mode network,” and “neuroimaging” as the most consistent terms associated with garnering the most citations from 2006 to 2015 (Yeung et al., 2017b). From 2012 to 2015, the terms “melatonin,” “microglia,” and “neurofibrillary tangle” each also emerged as high impact (Yeung et al., 2017b). Although direct comparison to present findings is limited, as the algorithm used here considered only single words, these terms' performance from 2014 to 2018 did not distinguish them as exceptionally high-impact associated: “*autism*” (1.08), “*melatonin*” (−1.45), “*meta-analysis*” (1.18), “*microglia*” (0.08), “*neurofibrillary*” (1.01), and “*neuroimaging*” (0.49). However, all of those terms garnered more citations than would be expected (citation rate residuals > 0).

Such a gross overview of an entire field is sure to come with caveats. Although effort was made to ensure the selection of journals was representative, there could still be lurking biases within their composition. It should also be noted that the citation impact of an individual researcher's early work is not a reliable predictor of career persistence (Milojević et al., 2018).

The field of neuroscience has long ago outgrown informal assessments and conventional wisdom. Only by monitoring the content of publications can we maintain an objective perspective on the field's priorities. Given the discrepancies between the present findings and the few comparable analyses of the past, it would appear that was in vogue 1 year may be superseded quickly. Indeed, the correlation of a given term's log odds ratio in 2010 with that of 2018 was only R^2^ = 0.34. That this churn in the field's interest can occur within the span of a young researcher's training period should have major implications for career mentorship. Analyses like those presented here should be carried our regularly, and ideally, in the future with more rigorous, completeness, and sophistication.

## Data Availability

The datasets and analysis code used for this study are freely available upon request.

## Author Contributions

WK conceived of and carried out the analyses and wrote the resulting manuscript.

### Conflict of Interest Statement

The author declares that the research was conducted in the absence of any commercial or financial relationships that could be construed as a potential conflict of interest.
